# Detection and genetic characterization of enteric viruses in diarrhoea outbreaks from swine farms in Spain

**DOI:** 10.1186/s40813-023-00326-w

**Published:** 2023-06-22

**Authors:** Héctor Puente, Héctor Arguello, Martí Cortey, Manuel Gómez-García, Oscar Mencía-Ares, Lucía Pérez-Perez, Ivan Díaz, Ana Carvajal

**Affiliations:** 1 Departamento de Sanidad Animal, Facultad de Veterinaria, Universidad de León, León, Spain; 2 INDEGSAL, Universidad de León, León, Spain; 3 Departament de Sanitat i Anatomia Animals, Facultat de Veterinària, Universitat Autònoma de Barcelona, Bellaterra, Spain; 4 IRTA, Programa de Sanitat Animal, Centre de Recerca en Sanitat Animal (CReSA), Campus de la Universitat Autònoma de Barcelona, Bellaterra, Spain; 5 Unitat Mixta d’investigació IRTA-UAB en Sanitat Animal, Centre de Recerca en Sanitat Animal (CReSA), Campus de la Universitat Autònoma de Barcelona, Bellaterra, Spain; 6 WOAH Reference Laboratory for Classical Swine Fever, IRTA-CReSA, Bellaterra, Spain

**Keywords:** Swine, Multiplex RT-PCR, Phylogenetic analysis, *Porcine astrovirus*, *Porcine kobuvirus*, *Porcine torovirus*, *Mammalian orthoreovirus*, *Porcine mastadenovirus*

## Abstract

**Background:**

The aim of this work was to study the prevalence and distribution of *Porcine astrovirus* (PAstV), *Porcine kobuvirus* (PKoV), *Porcine torovirus* (PToV), *Mammalian orthoreovirus* (MRV) and *Porcine mastadenovirus* (PAdV) as well as their association with widely recognized virus that cause diarrhoea in swine such as coronavirus (CoVs) and rotavirus (RVs) in diarrhoea outbreaks from Spanish swine farms. Furthermore, a selection of the viral strains was genetically characterized.

**Results:**

PAstV, PKoV, PToV, MRV and PAdV were frequently detected. Particularly, PAstV and PKoV were detected in almost 50% and 30% of the investigated farms, respectively, with an age-dependent distribution; PAstV was mainly detected in postweaning and fattening pigs, while PKoV was more frequent in sucking piglets. Viral co-infections were detected in almost half of the outbreaks, combining CoVs, RVs and the viruses studied, with a maximum of 5 different viral species reported in three investigated farms. Using a next generation sequencing approach, we obtained a total of 24 ARN viral genomes (> 90% genome sequence), characterizing for first time the full genome of circulating strains of PAstV2, PAstV4, PAstV5 and PToV on Spanish farms. Phylogenetic analyses showed that PAstV, PKoV and PToV from Spanish swine farms clustered together with isolates of the same viral species from neighboring pig producing countries.

**Conclusions:**

Although further studies to evaluate the role of these enteric viruses in diarrhoea outbreaks are required, their wide distribution and frequent association in co-infections cannot be disregard. Hence, their inclusion into routine diagnostic panels for diarrhoea in swine should be considered.

**Supplementary Information:**

The online version contains supplementary material available at 10.1186/s40813-023-00326-w.

## Background

Enteric diseases caused by viruses are highly prevalent and negatively impact productivity in pig production [1]. Coronaviruses (CoVs) and rotaviruses (RVs) are the most common and well-recognized viruses responsible for diarrhoea in pigs [2–4]. Among CoVs, *Transmissible gastroenteritis virus* (TGEV) and *Porcine epidemic diarrhoea virus* (PEDV), two *Alphacoronavirus*, have swept farms all over the world since their emergence in the last century [5, 6]. More recently, a recombinant virus between TGEV and PEDV, known as *Swine enteric coronavirus* (SeCoV), has been identified on European pig farms [7–10]. These three CoVs target the small intestine in pigs of all ages, shortening villi and inducing an undistinguishable diarrhoeic disease [3, 11]. Other two porcine CoVs, *Porcine delta coronavirus* (PDCoV) [12] and *Swine acute diarrhoea syndrome coronavirus* (SADS-CoV) [13, 14], an *Alphacoronavirus*, have been also recently described in diarrhoea outbreaks, although they have never been detected on European swine farms. RVs are also a major cause of acute gastroenteritis in swine worldwide with *Rotavirus A* (RVA) and *Rotavirus C* (RVC) as the most relevant species [3]. They are mainly involved in diarrhoea outbreaks in both nursing and weaned piglets although they can be detected in all stages in porcine production [3].

In addition, several other swine enteric viruses such as *Porcine astrovirus* (PAstV) [15–20], *Porcine kobuvirus* (PKoV) [16, 21–24], *Porcine torovirus* (PToV) [25–29], *Mammalian orthoreovirus* (MRV) [30–34] or *Porcine mastadenovirus* (PAdV) [1, 35–37] have been detected in pigs with diarrhoea worldwide (Table 1), although their etiological role remains unclear. These viruses are frequently identified in co-infections with CoVs and RVs [4, 38–45], and are also commonly detected in healthy animals [39, 46–50]. The enteropathogenicity of PAstV, MRV and PAdV has been demonstrated on experimentally infected conventional piglets [51–55], but to the best of the authors’ knowledge, no experimental challenge has been reported for PKoV and PToV.

**Table 1:** Main characteristics of porcine enteric viruses with well-recognized or unclear involvement in the etiology of diarrhoea outbreaks on swine farms

Virus	Genus	Family	Enveloped virus	Viral genome structure	Genome size (Kb)	Identified in swine samples
*Well-recognized viruses responsible for diarrhoea outbreaks on swine farms*						
PEDV	*Alphacoronavirus*	*Coronaviridae*	Yes	RNA single-stranded positive sense	28–30	[2, 58, 72]
TGEV	*Alphacoronavirus*	*Coronaviridae*	Yes	RNA single-stranded positive sense	28–30	[72–74]
SeCoV	*Alphacoronavirus*	*Coronaviridae*	Yes	RNA single-stranded positive sense	28–30	[7–10]
PDCoV	*Deltacoronavirus*	*Coronaviridae*	Yes	RNA single-stranded positive sense	28–30	[12, 75, 76]
SADS	*Alphacoronavirus*	*Coronaviridae*	Yes	RNA single-stranded positive sense	28–30	[13, 14, 77–79]
RVA	*Rotavirus*	*Sedoreoviridae*	No	RNA double-stranded segmented (11)	18–19	[3, 80, 81]
RVC	*Rotavirus*	*Sedoreoviridae*	No	RNA double-stranded segmented (11)	18–19	[3, 80, 82]
*Enteric viruses with an unclear role in diarrhoea outbreaks on swine farms*						
PAstV	*Astrovirus*	*Astroviridae*	No	RNA single-stranded positive sense	6.4–7.3	[15–20]
PKoV	*Kobuvirus*	*Picornaviridae*	No	RNA single-stranded positive sense	8.2–8.3	[16, 21–24]
PToV	*Torovirus*	*Tobaniviridae*	Yes	RNA single-stranded positive sense	25–30	[25–29]
MRV	*Orthoreovirus*	*Spinareoviridae*	No	RNA double-stranded segmented (10)	23–24	[30–34]
PAdV	*Mastadenovirus*	*Adenoviridae*	No	DNA double-stranded lineal	32–34	[1, 35–37]

MRV: *Mammalian orthoreovirus*, PAdV: *Porcine mastadenovirus*, PAstV: *Porcine astrovirus*, PEDV: *Porcine epidemic diarrhoea virus*, PDCoV: *Porcine deltacoronavirus*, PKoV: *Porcine kobuvirus*, PToV: *Porcine torovirus*, RVA: *Rotavirus A*, RVC: *Rotavirus C*, SADS: *Swine acute diarrhoea syndrome coronavirus*, SeCoV: *Swine enteric coronavirus*, TGEV: *Transmissible gastroenteritis virus*

The aim of this work was to study the prevalence and distribution of PAstV, PKoV, PToV, MRV and PAdV in diarrhoea outbreaks on swine farms in Spain, as well as to identify their association with widely recognized virus that cause diarrhoea, such as CoV and RV. In addition, a selection of the viral strains was genetically characterized. Altogether, our results provide information on the aetiology of enteric disease in swine that can be used to devise and implement appropriate diagnostic and control strategies.

## Results

### Prevalence and distribution of individual swine enteric viruses in diarrhoea outbreaks in Spain

Globally, 160 out of 206 (77.7%) farms were positive to at least one of the enteric viruses tested. PAstV was the most frequently detected virus (48.5%; n = 100) followed by PKoV (27.2%; n = 56), PEDV (19.9%; n = 41), PAdV (14.1%; n = 29), RVA (13.6%; n = 28), PToV (11.2%; n = 23), RVC (6.3%; n = 13) and MRV (4.4%; n = 9). TGEV or SeCoV were not detected in any of the investigated farms.

**Fig. 1:**
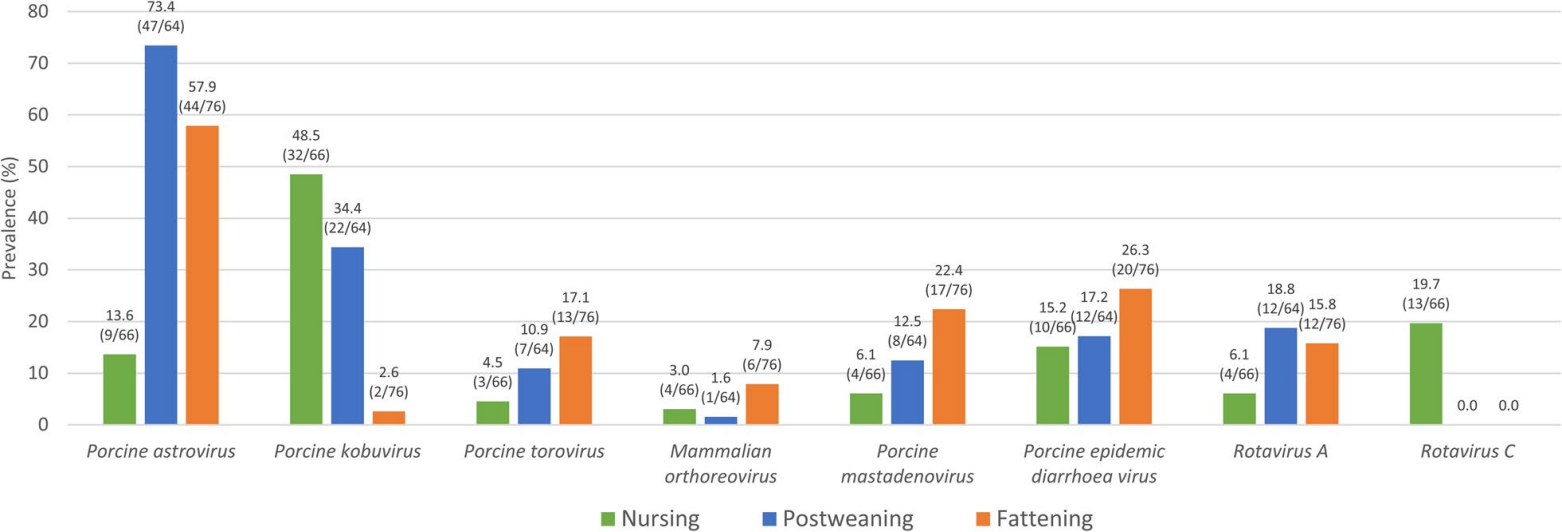
Prevalence of positive diarrhoea outbreaks to each of the different investigated enteric viruses according to the age of the affected animals. Out of the 206 investigated diarrhoea outbreaks, 66 affected nursing piglets (< 21 days), 64 postweaning-growing piglets (21–70 days) and 76 fattening pigs (> 70 days)

The prevalence of positive diarrhoea outbreaks (a positive result for at least one of the tested viruses) was similar (*p* = 0.787) among nursing (75.8%, 50 out of 66), postweaning (76.6%, 49 out of 64) and fattening periods (80.3%, 61 out of 76). However, different age distribution patterns were observed for each of the investigated viruses (Fig. 1). Detection of PAstV and RVA was significantly lower in nursing diarrhoea outbreaks as compared with postweaning (*p* < 0.001 and *p* = 0.025, respectively) and fattening outbreaks (*p* < 0.001 and *p* = 0.036, respectively). In contrast, PKoV were more prevalent in nursing and postweaning than in fattening (*p* < 0.001), while RVC were only detected in diarrhoea outbreaks affecting lactating piglets. Finally, PToV and PAdV were more frequently detected in fattening outbreaks as compared to nursing (*p* = 0.016 and *p* = 0.005, respectively). A similar trend was observed for PEDV and MRV although without statistical significance.

In addition, it was evidenced that most of the investigated outbreaks (50.0%; 103 out of 206) occurred during the first trimester as compared with the rest of the year (*p* < 0.001). PEDV was more commonly detected during the winter (January to March) (*p* = 0.011) while no associated seasonality could be confirmed for any of the other investigated enteric viruses (Additional file 1).

**Fig. 2:**
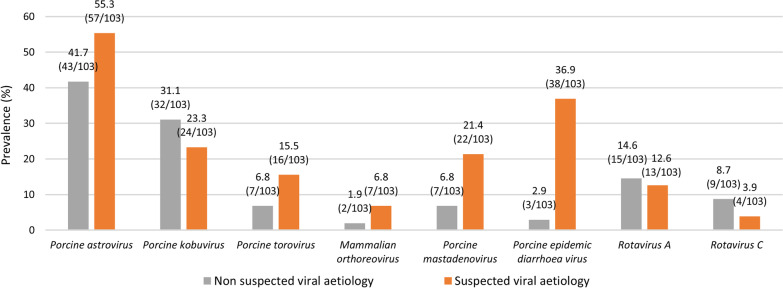
Prevalence of positive diarrhoea outbreaks to each of the different investigated enteric viruses according to the epidemiological and clinical appearance of the outbreaks (non-suspected—n = 103- or suspected—n = 103-viral involvement)

Based on clinical criteria, the diarrhoea outbreaks included in this research were classified by the veterinarian responsible for the farm into outbreaks with or without a suspected viral aetiology. The risk of a positive result to at least one the tested viruses was higher in viral suspected outbreaks (OR = 2.52, CI 95% 1.27–5.04) as compared with those without viral suspicion (85.4% vs. 69.9%). Accordingly, the prevalence of each of the investigated viruses was higher in outbreaks with suspected viral aetiology, with the exception of PKoV, RVA and RVC (Fig. 2), although statistical differences were only demonstrated for PEDV (*p* < 0.001) and PAdV (*p* = 0.002).

### Prevalence and distribution of viral co-infections in diarrhoea outbreaks in Spain

In 69 out of the 206 analysed outbreaks (33.5%) a single viral aetiological agent was detected, while 56 (27.2%) presented a co-infection of two viruses at the same time and 35 (17.0%) were positive for more than two (up to 5 different virus species simultaneously detected on three different farms). Detection of a single viral agent was more frequent in diarrhoea outbreaks in lactation as compared with postweaning (26.6%, 17 out of 64, *p* = 0.019) and fattening (28.9%, 22 out of 76, *p* = 0.031). On the contrary, outbreaks with two or more co-infecting viruses were more prevalent in postweaning (50.0%, 32 out of 64, *p* = 0.017) and fattening (51.3%, 39 out of 76, *p* = 0.009) as compared with nursing (30.3%, 20 out of 66). Besides, outbreaks presenting two or more co-infecting viruses were more frequent when viral aetiology was suspected according to the criteria of the veterinarian responsible for the farm (55.3% vs. 33.0%, *p* = 0.001).

As shown in Additional file 2, PAstV and PKoV were detected in almost 30% of the positive outbreaks as a single infection while PToV, MRV and PAdV were detected in over 90% of positive outbreaks co-infecting with PEDV, RVs or other enteric viruses. The most frequent viral combinations (top 8) are shown in Table 2. Once again, a clear age-dependent distribution was observed with co-infections including PAstV more prevalent in postweaning and fattening outbreaks and co-infections with PKoV recorded among the top 3 in nursing outbreaks.

**Table 2:** Most common viral co-infections reported (top 8) in the investigated diarrhoea outbreaks according to the age of the affected animals

Age of the affected animals (number of investigated outbreaks)											
Nursing (n = 66)			Postweaning (n = 64)			Fattening (n = 76)			Total (n = 206)		
Rank	Viruses	Prevalence (%)	Rank	Viruses	Prevalence (%)	Rank	Viruses	Prevalence (%)	Rank	Viruses	Prevalence (%)
1	PKoV + PEDV	7.6	1	PAstV + PKoV	32.8	1	PAstV + PAdV	18.4	1	PAstV + PKoV	12.6
2	PKoV + RVC	7.6	2	PAstV + PEDV	15.6	2	PAstV + PToV	13.2	2	PAstV + PEDV	11.2
3	PKoV + RVA	4.5	3	PKoV + RVA	12.5	3	PAstV + PEDV	13.2	3	PAstV + PAdV	10.7
4	PAstV + PEDV	4.5	4	PAstV + RVA	10.9	4	PAstV + RVA	7.9	4	PAstV + RVA	9.7
5	PAstV + PKoV	4.5	5	PAstV + PToV	10.9	5	PAstV + MRV	7.9	5	PAstV + PToV	8.7
6	PAstV + RVA	3.0	6	PAstV + PAdV	9.4	6	PToV + PEDV	5.3	6	PKoV + RVA	5.3
7	PKoV + PToV	3.0	7	PKoV + PEDV	7.8	7	PToV + RVA	5.3	7	PKoV + PEDV	4.9
8	PToV + PEDV	3.0	8	PAstV + PToV	7.8	8	PToV + PAdV	3.9	8	PToV + PEDV	4.4

PAdV: *Porcine mastadenovirus*, PAstV: *Porcine astrovirus*, PEDV: *Porcine epidemic diarrhoea virus*, PKoV: *Porcine kobuvirus*, PToV: *Porcine torovirus*, RVA: *Rotavirus A* and RVC: *Rotavirus C*

**Fig. 3:**
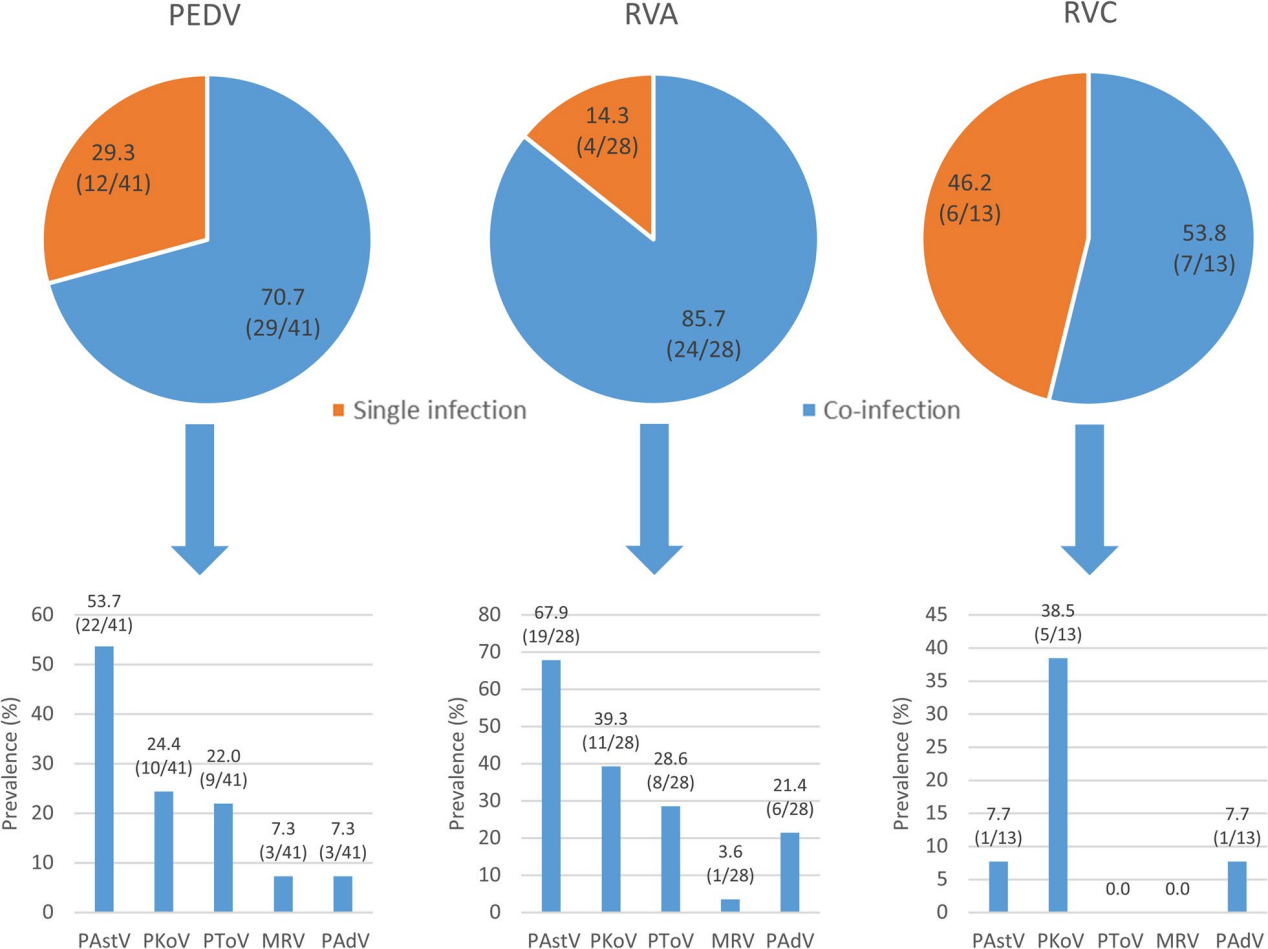
Detection of each of the different investigated enteric viruses (*Porcine astrovirus* or PAstV, *Porcine kobuvirus* or PKoV, *Porcine torovirus* or PToV, *Mammalian orthoreovirus* or MRV and *Porcine adenovirus* or PAdV) in positive outbreaks involving well-recognized viruses that cause diarrhoea (*Porcine epidemic diarrhoea virus* or PEDV—n = 41-, *Rotavirus A* or RVA—n = 28- and *Rotavirus C* or RVC—n = 13-)

The viruses most frequently associated with PEDV, RVA and RVC are shown in Fig. 3. PAstV was identified in more than 50% of the PEDV and RVA outbreaks while RVC mainly co-infects with PKoV, probably as a consequence of the age dependent distribution of these infections. Comparison of the prevalence of each of the investigated viruses between PEDV, RVA and RVC positive and negative outbreaks reveal that PToV were more commonly detected in PEDV and RVA positive outbreaks (*p* = 0.021 and *p* = 0.005, respectively) while PAstV detection was more common in RVA positive farms (*p* = 0.027).

### Sequencing and phylogenetic analysis of PAstV, PKoV and PToV

By using a next generation sequencing (NGS) in 14 pooled faecal samples, we identified 10 RNA virus species from six different genera: PAstV2 (6), PAstV3 (7), PAstV4 (10), PAstV5 (7), PkoV (8), PToV (5), PEDV (10), RV (2·RVA and 2 RVC) and MRV (1) (Additional file 3). From this array of viruses, the whole or almost whole genome was sequenced in 24, including PAstV2 (5), PAstV3 (1), PAstV4 (8), PAstV5 (2), PKoV (3), PToV (1) and PEDV (4).

**Fig. 4:**
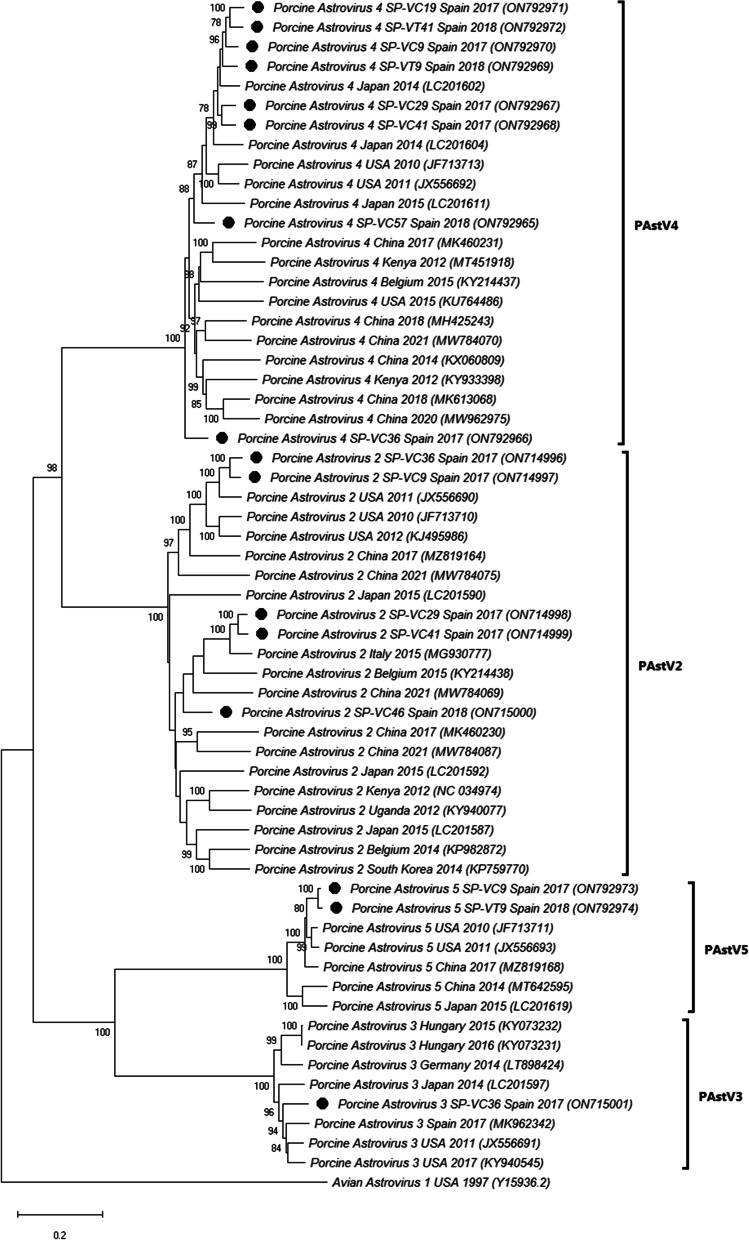
Neighbor-joining phylogenetic tree based on the p-distance among the nucleotide sequences of the complete genomes for PAstV. Along the branches, percentage of bootstrap values based on 10,000 replicates (only values equal or larger than 70% are shown). The filled circles identified the isolates sequenced in this research. GenBank accession number, country and year of the outbreak are also shown below the strains. The PAstV lineages referred in the text are included on the right of the tree. Scale bars indicate nucleotide substitutions per site

Four species of PAstV (PAstV2 to PAstV5) were detected in the investigated outbreaks on Spanish farms, being PAstV4 and PAstV2 the most represented. The nucleotide sequence identities between PAstV2 and PAstV4 strains recovered in our research were 77.2–95.2% and 87.2–93.7% respectively, while its comparison with previously described sequences provided nucleotide identities of 68.7–91.0% for PAstV2, 87.6–89.1% for PAstV3, 73.4–91.8% for PAstV4 and 83.3–94.4% for PAstV5. Most of the Spanish PAstV sequences clustered together with North American strains of the same lineage, although PAstV4 clustered with sequences from Japan and some PAstV2 with sequences from Europe and China (Fig. 4).

**Fig. 5:**
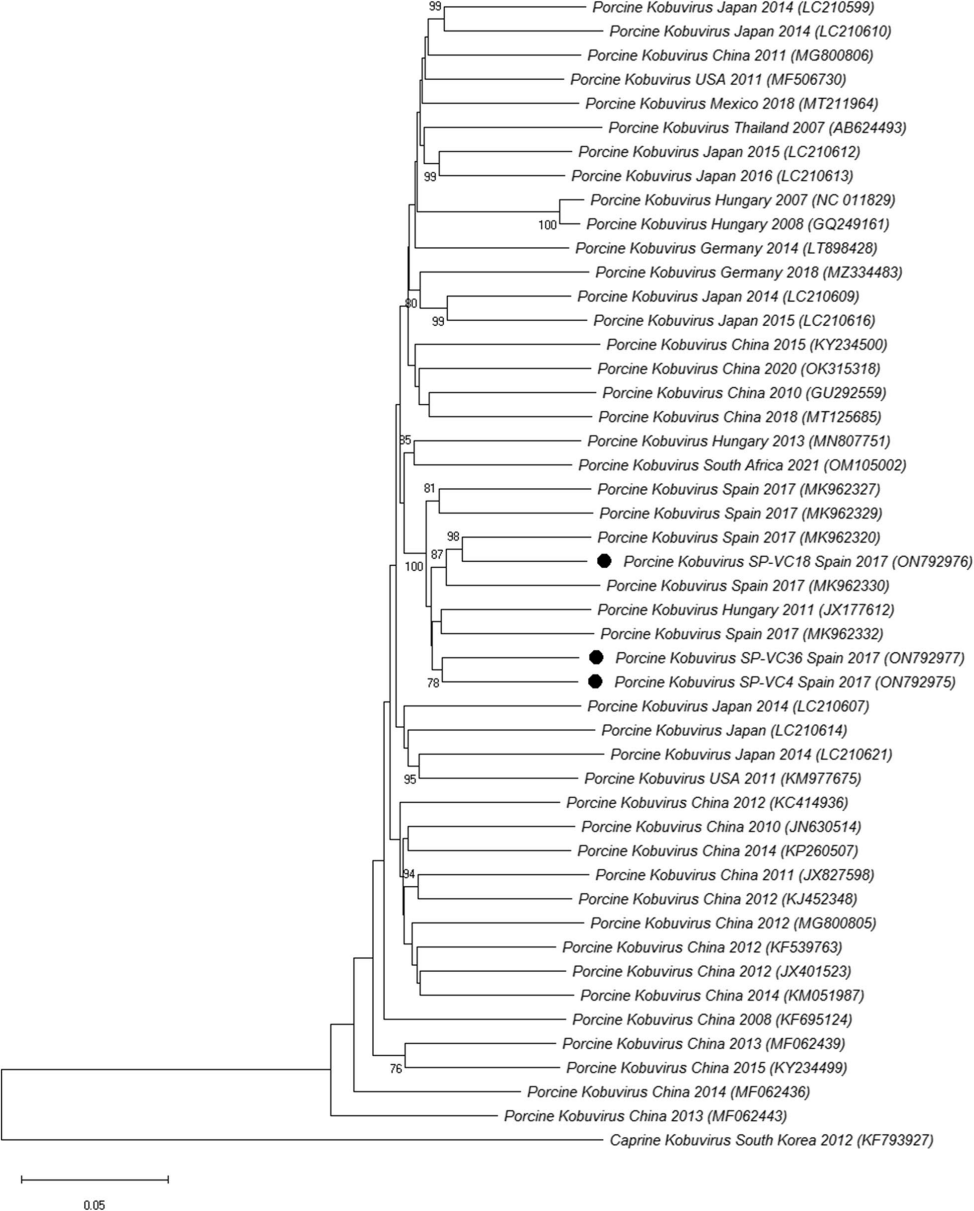
Neighbor-joining phylogenetic tree based on the p-distance among the nucleotide sequences of the complete genomes for PKoV. Along the branches, percentage of bootstrap values based on 10,000 replicates (only values equal or larger than 70% are shown). The filled circles identified the isolates sequenced in this research. GenBank accession number, country and year of the outbreak are also shown below the strains. Scale bars indicate nucleotide substitutions per site

Regarding the phylogenetic analysis of PKoV (Fig. 5), the three sequences obtained in our study clustered together with previously reported Spanish and Hungarian isolates. The nucleotide sequence identity was 90.3–91.4% among the Spanish isolates identified in this study and 87.2–91.9% with the previously reported whole genome PKoV sequences.

**Fig. 6:**
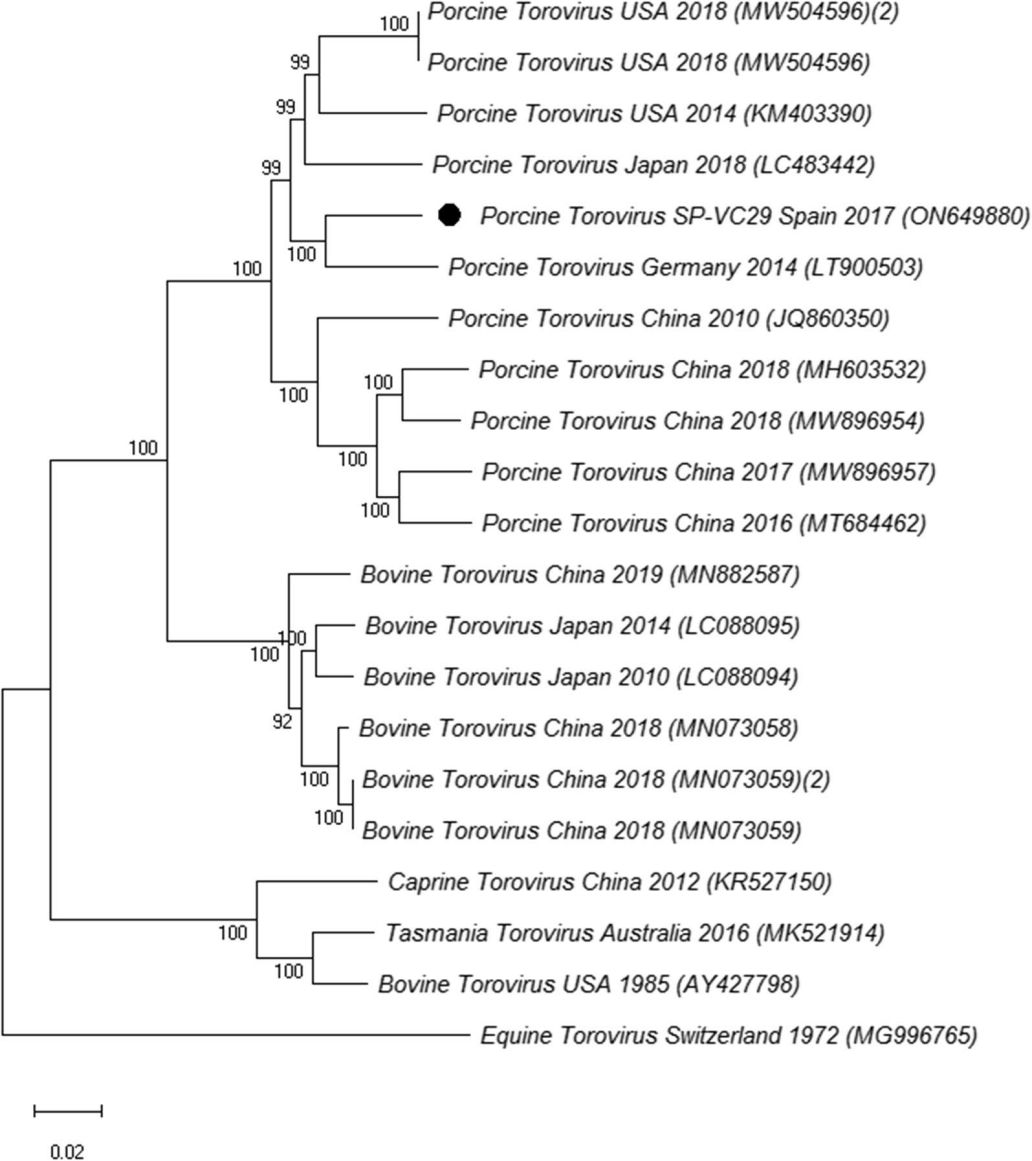
Neighbor-joining phylogenetic tree based on the p-distance among the nucleotide sequences of the complete genomes for PToV. Along the branches, percentage of bootstrap values based on 10,000 replicates (only values equal or larger than 70% are shown). The filled circles identified the isolates sequenced in this research. GenBank accession number, country and year of the outbreak are also shown below the strains. Scale bars indicate nucleotide substitutions per site

Finally, a single PToV complete genome was obtained. When compared with previously described PToV its nucleotide identity was 88.6–94.1%, but was lower when compared with torovirus sequences from other animal species (77.3–87.9%). This Spanish PToV sequence clustered together with viral sequences from Europe, particularly Germany, but also with sequences from the USA and Japan (Fig. 6).

## Discussion

The enteropathogenic role of PAstV, PKoV, PToV, MRV and PAdV is still controversial and has not been evidenced in a number of case–control studies comparing detection ratios in diarrhoeic and healthy pigs [39, 46–50]. Taking this fact into account, we investigated the prevalence of these enteric viral species focusing on diarrhoea outbreaks and looking into their association with well-recognized viral enteropathogens as CoVs (PEDV, TGEV and SeCoV) and RVs (RVA and RVC). The design of our study does not allow to draw conclusions about the role of these viruses in the aetiology of enteric disease, but it aims to increase knowledge about their frequency, distribution and co-infections on farms with enteric problems.

Our results confirmed that PAstV and PKoV infections are highly prevalent on European farms, as previously proposed by a number of studies [19, 46, 49, 50, 56]. PAstV was the most frequent enteric virus in our research, detected in almost half of the outbreaks, followed by PKoV, detected in more than 25% of the investigated farms. In agreement with a previous report in Hungary [49] PAdV was less common (14.1% of positive outbreaks) while PToV was only identified in 11.2% of the investigated farms in spite of the broad distribution of this virus, evidenced by another Spanish study which estimated over 99% of seropositive animals among pigs older than 11 weeks of age [57]. Finally, MRV was the least frequently detected enteric virus (4.4%) among those with an unclear aetiological role in diarrhoea outbreaks. To the authors knowledge there are no previous reports on the prevalence of MRV on European swine farms. Regarding well-recognized enteropathogenic viruses, neither TGEV nor SeCoV were detected. PEDV was identified in 19.9% of the investigated diarrhoea outbreaks and the prevalence of RVA and RVC positive outbreaks was 13.6 and 6.3%, respectively.

Three age distribution patterns were observed among the investigated enteric viruses. Similarly to previous studies in China [16, 47], Canada [44] and several European countries [38, 49, 50], PKoV were more prevalent in nursing and postweaning piglets as compared with fattening stages. A similar distribution has been reported for RVC [3] and was confirmed in our research, with no positive RVC outbreak occurring after weaning. In contrast, PAstV and RVA showed a significant increase in their prevalence in weaned pigs as compared to nursing pigs. Previous studies on European swine farms have reported a similar trend for PAstV [46, 49, 50]. As described for endemic RVA on swine farms [1], the decline of specific maternal antibodies could explain the increase in prevalence of PAstV in the postweaning period. Finally and in line with previous studies [28, 49], PToV and PAdV were identified more frequently in diarrhoea outbreaks during the fattening period.

A seasonal distribution was only demonstrated for PEDV positive outbreaks which were more common during the coldest months of the year (January–March). Low temperatures allowing for CoV survival in the environment and facilitating indirect transmission may explain seasonal variation in PEDV outbreaks [58, 59]. Moreover, it has been proposed that the exposure to low ambient temperatures may enhance PEDV replication through up-regulated heat shock protein Hsp70 in the pig intestine, contributing to PEDV seasonality [60]. In contrast, no seasonality has been described for porcine RV [3].

Interestingly, veterinary clinical criteria suggesting a presumptive viral aetiology was associated with the viral detection using molecular methods. This was particularly evident for PEDV positive outbreaks but also for PAdV and to a lesser extent for PAstV, PToV or MRV. In contrast, RVA, RVC and PKoV were detected mainly in non-viral suspected outbreaks. The fact that RV infections are endemic in swine populations and frequently associated with other diarrhea-producing agents such as enterotoxigenic *Escherichia coli* or *Clostridium perfringens* type A [3, 61, 62] could complicate its recognition by practitioners.

Co-infections were relatively frequent with two or more viruses detected in almost 40% of the investigated outbreaks. This result is in line with previous studies on European farms [16, 49, 63] reporting a high frequency of co-infections involving several enteric viruses in pigs with diarrhoea. A higher proportion of viral enteric co-infections, close to 70%, has been described on swine farms from Asia [4, 40]. In agreement with previous studies conducted in Europe [49], China [4, 47] or Canada [44], the number of different virus species detected within an outbreak increased with the age of the affected pigs. Hence, single infections were more common in nursing piglets while simultaneous co-infections with up to five viruses were observed in postweaning and fattening pigs. Many factors associated with weaning such as dietary changes, placement into a new and more contaminated environment, mixing of pigs or loss of passive immunity can favor the infection by different viral species. Also, it has been proposed that the maturation of the gut associated immune system after weaning may prevent the development of enteric disease associated to single infections for those enteric viruses with a moderate enteropathogenicity [64].

The most common co-infections varied depending on the age or life stage of the affected pigs. As expected from the observed patterns of age distribution, co-infections involving PKoV were the most prevalent in lactating piglets (27.2%) while co-infections with PAstV predominated in postweaning and fattening outbreaks (87.4% and 60.6%, respectively). Although it has been recently proposed that PEDV and PKoV might play some synergetic role [65], no significant association between both viruses was observed in our research. The fact that most of the PEDV outbreaks occurred in postweaning or fattening pigs when PKoV is less prevalent may explain this result. In contrast, PToV shedding was more frequent in PEDV and RVA positive outbreaks. Although there is very little information known about PToV infections, it is worth mentioning that the first report of PToV in the United States was in a PEDV positive pig [66]. In addition, an increase in the prevalence of PAstV shedding was observed in RVA positive outbreaks. Further research is required to decipher the interactions between enteric viruses which may play a primary or secondary role in the aetiology of enteric disease in pigs.

Given the high prevalence of viral co-infections detected, the use of conventional PCR for viral detection is a major limitation of our research. Quantitative approaches based on qPCR or next generation sequencing (NGS) would have allowed for viral load estimations. As proposed by Cortey et al. [63] the determination of the relative abundance of different viral species in co-infections makes it possible to identify the predominant virus which can most likely be proposed as the primary etiological agent.

Finally, the study involved the fine tune genomic characterization of a selection of samples to obtain the complete sequence of part of these viruses. Phylogenetic analysis revealed that the majority of the PAstV strains were classified as PAstV4 (50.0%, 8/16) and PAstV2 (31.2%, 5/16), indicating that these two PAstV species are the most prevalent in swine with diarrhoea in Spain, as it has been described in several European countries [46, 50, 67], in North America [68] and in Asia [39, 69, 70]. PKoV phylogenetic analyses revealed that all available Spanish complete sequences (including the three identified in our study) were included in a single local cluster together with one Hungarian strain and well differentiated from other European isolates [38]. Finally, the single PToV whole genome sequence obtained in this research clustered together with other PToV isolates from Europe, America or Asia [25, 26].

## Conclusions

Although the design of our study does not allow for the evaluation of the role of PAstV, PKoV, PToV, MRV or PAdV as causative agents of diarrhoea in pigs, we have demonstrated a high prevalence of these enteric viruses in diarrhoea outbreaks, particularly for PAstV and PKoV detected in almost 50% and 30% of the investigated farms. The fact that these enteric viruses are frequently involved in co-infections among them and also with well-recognized viral enteropathogens as CoVs or RVs make necessary further studies for surveillance and characterization as well as investigations to evaluate their pathogenic potential in both experimental and natural infections. Once these studies are available, the inclusion of these viruses into the routine diagnostic panels for diarrhoea in swine should be considered.

## Methods

### Sample collection and nucleic acid extraction

The study was conducted between January 2017 and October 2020 on 206 Spanish swine farms with diarrhoea outbreaks affecting nursing piglets (< 21 days) (66 farms), postweaning-growing (21–70 days) (64 farms) or fattening pigs (> 70 days) (76 farms). From each farm, two to six individual fecal samples were submitted for diagnostic purposes to the Infectious Diseases Unit of the Animal Health Department of the University of León (Spain). The veterinarian responsible for the farm classified the outbreak as viral or non-viral suspicion, according to criteria such as speed of progression of the disease or response to antimicrobials.

Individual fecal samples were mixed to prepare one pooled sample per farm that was diluted 1:2 (v/v) in sterile phosphate buffered saline (PBS), homogenized by vortex mixing and centrifuged for 10 min at 20,000 g. The nucleic acid was extracted from 140 µl of the supernatant using QIAMP Viral RNA and DNA Mini Kit (QIAGEN), following the manufacturer’s instructions, and stored at − 80 °C until use.

### Molecular detection of porcine enteric viruses

Three duplex and one triplex RT-PCRs were carried out using Verso 1-Step RT-PCR ReddyMix kit (Thermo Scientific) for the detection of PEDV, TGEV, SeCoV (simultaneous detection of S gene of PEDV and N gene of TGEV), RVA, RVC, PToV, PAstV, PKoV, MRV and PAdV (Table 3). The reactions were conducted under the following conditions: 50 °C for 30 min, 95 °C for 2 min, 45 cycles at 95 °C for 20 s, 50 °C for 30 s, and 72 °C for 1 min and 30 s, followed by a final extension step at 72 °C for 10 min.

**Table 3:** Primer sets used for the detection of porcine enteric viruses by multiplex RT-PCR

Virus	Primer	Sequence (5'–3')	Target	Size (bp)	References
*RT-PCR multiplex 1*					
TGEV	TGEV-TA	GATGGCGACCAGATAGAAGT	Nucleocapsid gene	612	[83]
	TGEV-TB	GCAATAGGGTTGCTTGTACC			
PToV	ToV-F	ATTGCTTATTGGTGGCTTCC	Spike gene	464	This study
	ToV-R	GGCTACTCAAACTTAACACT			
PAstV	AstV-F	ATGTCTTTGGGATGTGG	RdRp gene	334	This study
	AstV-R	TTTGGTCCTCCCCTCCAAAG			
*RT-PCR multiplex 2*					
PEDV	PEDV-F	TTCTGAGTCACGAACAGCCA	Spike gene	651	[84]
	PEDV-R	CATATGCAGCCTGCTCTGAA			
PKoV	KV-F1	CGGGACTGGTTTGGAGGAACA	Leader gene	446	[24]
	KV-R	CTCTATCAAGCAGTACATGG			This study
*RT-PCR multiplex 3*					
RVC	RVC-VP7-F	GCTGTCTGACAAACTGGTC	Viral protein 7 gene	1061	[85]
	RVC-VP7-R	GCCACATGATCTTGTTTACGC			
MRV	MRV-L1-F	CTCGTGAAATGGCGAATGTG	RdRp Lambda 3 gene	638	This study
	MRV-L1-R	TAGACTCACGCTGACCGTCC			
*RT-PCR multiplex 4*					
RVA	RVA-NSP2-F	GGCTTTTAAAGCGTCTCAGTC	Non-structural protein 2 gene	1059	[86]
	RVA-NSP2-R	GGTCACATAAGCGCTTTCTATTC			
PAdV	AdV-PALF	GATGTCATGGAYAACGTCAAC	Hexon gene	612	[36]
	AdV-PARF	CACGGAGGAGTCRAACTGGATG			

MRV: *Mammalian orthoreovirus*, PAdV: *Porcine mastadenovirus*, PAstV: *Porcine astrovirus*, PEDV: *Porcine epidemic diarrhoea virus*, PKoV: *Porcine kobuvirus*, PToV: *Porcine torovirus*, RVA: *Rotavirus A*, RVC: *Rotavirus C* and TGEV: *Transmissible gastroenteritis virus*

The RT-PCR products were visualized on a 1.5% agarose gel containing RedSafe Nucleic Acid Staining Solution (iNtRON Biotechnology, Inc.) and compared with expected lengths (Table 3).

### Sequencing and phylogenetic analysis

A total of 14 pooled faecal samples were chosen for sequencing based on their RT-PCR results (samples with the highest number of different viruses detected). From each pooled sample, total RNA was extracted using a TRIzol LS reagent (Thermo Scientific) protocol. The total RNA extraction was directly sequenced at the Genomics Bioinformatics Service (SGB) of the Autonomous University of Barcelona (UAB), without using any primer or amplification step. NGS was carried out using an Illumina Miseq Platform. RNA virus sequences were obtained from NGS outputs applying a tailor-made, virus-specific script developed by Cortey et al. [63]. Subsequently, a database for every RNA virus species identified was constructed by downloading the available complete genome sequences from GenBank. Whole genome (> 90%) sequences were deposited in NCBI GenBank with the accession numbers ON649880, ON714996–ON715001 and ON792965–ON792977. Four whole genome sequences of PEDV also recovered in this work were already deposited with accession numbers MN692786, MN692787, MN692789 and MN692791 [8]. Finally, the sequences obtained were aligned and phylogenetically compared with the datasets constructed for every complete or near-complete genome identified. The phylogenetic relationships among sequences were analyzed using the software MEGA11 [71], by means of a Neighbor-joining (NJ) algorithm, using the model of maximum composite likelihood and 10,000 bootstrap replicates to estimate the confidence of the internal branches of the trees.

### Statistical analysis

Statistical analyses of data were performed by Fisher’s exact and ANOVA tests using Epi Info™. A two-sided *p* value of ≤ 0.05 was considered to indicate statistical significance.

## Supplementary Information

**Additional file 1**: Percentage of positive detection to each of the different investigated enteric viruses in diarrhoea outbreaks occurring between January 2017 and December 2019.

**Additional file 2**: Distribution of single infections and co-infections for* Porcine astrovirus* (PAstV),* Porcine kobuvirus* (PKoV),* Porcine torovirus* (PToV),* Mammalian orthoreovirus* (MRV) and* Porcine mastadenovirus* (PAdV) with well recognized pathogenic enteric viruses (*Porcine epidemic diarrhoea virus* or PEDV,* Rotavirus A* or RVA and* Rotavirus C* or RVC) and potential pathogenic enteric viruses (PAstV, PKoV, PToV, MRV and PAdV) in the investigated diarrhoea outbreaks (n = 206)

**Additional file 3**: Summary of the results of next generation sequencing (NGS) in 14 selected faecal samples. C, consensus sequence (whole genome obtained or near complete genome, >90%); P, partial sequence (less than 90% genome covered) and T, traces (less than 10% of the genome covered). N is the number of viruses detectedper sample. An asterisk indicates PEDV genomes that were already published [8].

## Data Availability

Data are available in the GenBank database and by direct contact with the correspondence author.

## Abbreviations

CI: Confidence interval
CoV: Coronavirus
MRV: *Mammalian orthoreovirus*
NGS: Next generation sequencing
PAdV: *P* *orcine mastadenovirus*
PAstV: *Porcine astrovirus*
PBS: Phosphate-buffered saline
PEDV: *Porcine epidemic diarrhoea virus*
PDCoV: *Porcine deltacoronavirus*
PKoV: *Porcine kobuvirus*
PToV: *Porcine torovirus*
RNA: Ribonucleic acid
RT-PCR: Reverse transcription-polymerase chain reaction
RV: Rotavirus
RVA: *Rotavirus A*
RVC: *Rotavirus C*
SADS: *Swine acute diarrhoea syndrome coronaviru*S
SeCoV: *Swine enteric coronavirus*
TGEV: *Transmissible gastroenteritis virus*
