# Supplementary figures and images for: Detection and genetic characterization of enteric viruses in diarrhoea outbreaks from swine farms in Spain
Source: Porcine Health Manag. 2023 Jun 22;9:29. doi: 10.1186/s40813-023-00326-w (PMC10286445; doi:10.1186/s40813-023-00326-w)

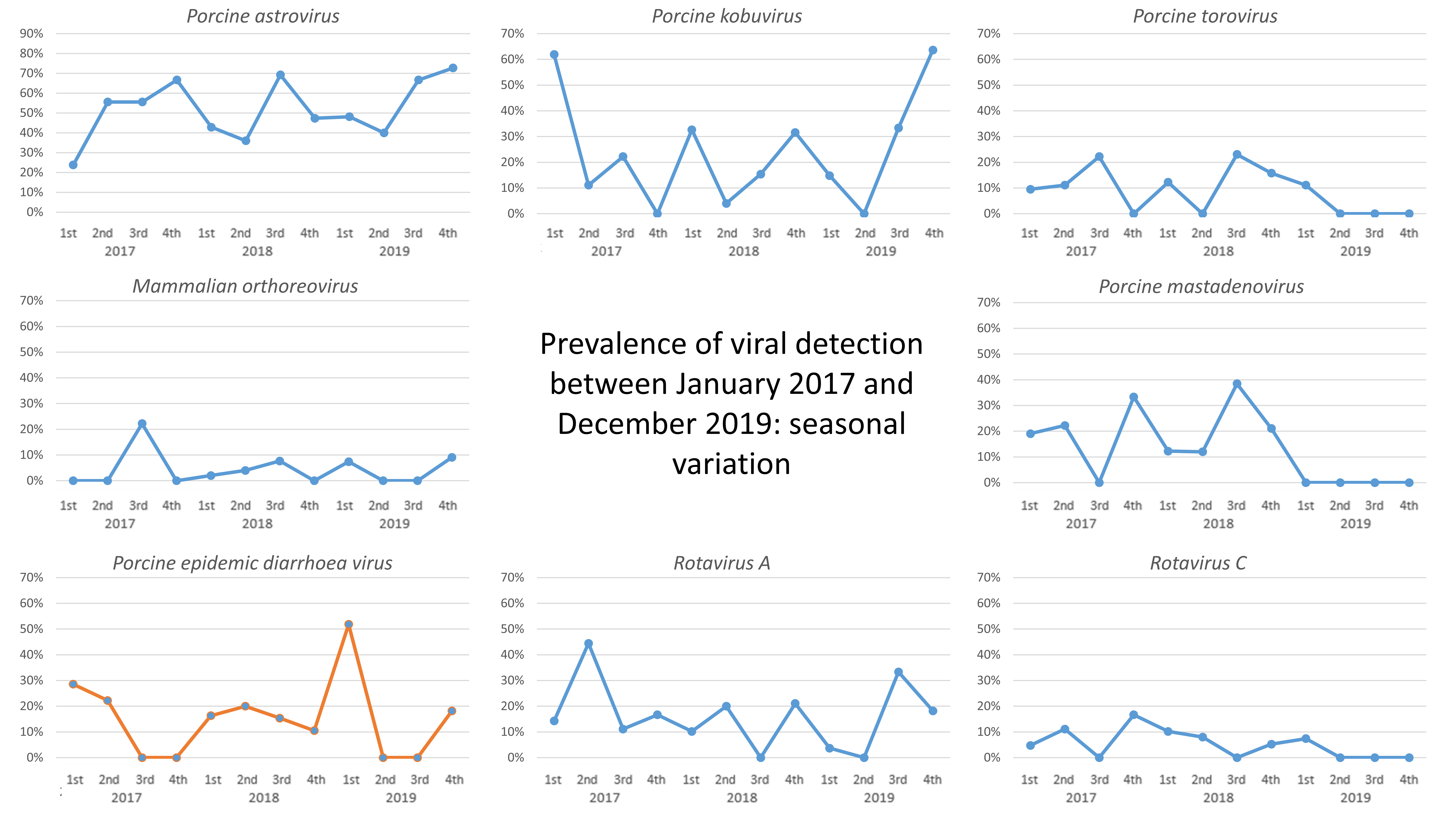

Supplement: Supplementary file 1 — Additional file 1: Percentage of positive detection to each of the different investigated enteric viruses in diarrhoea outbreaks occurring between January 2017 and December 2019. [file 40813_2023_326_MOESM1_ESM.tif]
