# Supplementary material for: Detection and genetic characterization of enteric viruses in diarrhoea outbreaks from swine farms in Spain
Source: Porcine Health Manag. 2023 Jun 22;9:29. doi: 10.1186/s40813-023-00326-w (PMC10286445; doi:10.1186/s40813-023-00326-w)
Supplement: Supplementary file 2 — Additional file 2: Distribution of single infections and co-infections for Porcine astrovirus (PAstV), Porcine kobuvirus (PKoV), Porcine torovirus (PToV), Mammalian orthoreovirus (MRV) and Porcine mastadenovirus (PAdV) with well recognized pathogenic enteric viruses (Porcine epidemic diarrhoea virus or PEDV, Rotavirus A or RVA and Rotavirus C or RVC) and potential pathogenic enteric viruses (PAstV, PKoV, PToV, MRV and PAdV) in the investigated diarrhoea outbreaks (n = 206) [file 40813_2023_326_MOESM2_ESM.docx]

**Additional file 2**: Distribution of single infections and co-infections for *Porcine astrovirus* (PAstV), *Porcine kobuvirus* (PKoV), *Porcine torovirus* (PToV), *Mammalian orthoreovirus* (MRV) and *Porcine adenovirus* (PAdV) with well-recognized pathogenic enteric viruses (*Porcine epidemic diarrhoea virus* or PEDV, *Rotavirus A* or RVA and *Rotavirus C* or RVC) and potential pathogenic enteric viruses (PAstV, PKoV, PToV, MRV and PAdV) in the investigated diarrhoea outbreaks (n = 206).

|  | **PAstV positive outbreaks (n = 100)** | **PKoV positive outbreaks (n = 56)** | **PToV positive outbreaks (n = 23)** | **MRV positive outbreaks (n = 9)** | **PAdV positive outbreaks (n = 29)** |
| --- | --- | --- | --- | --- | --- |
| Single infection | 28.0% | 32.1% | 4.3% | 0.0% | 6.9% |
| Co-infection with PAstV | **-** | 46.4% | 78.3% | 88.9% | 75.9% |
| Co-infection with PKoV | 26.0% | **-** | 30.4% | 11.1% | 20.7% |
| Co-infection with PToV | 18.0% | 12.5% | **-** | 33.3% | 17.2% |
| Co-infection with MRV | 8.0% | 1.8% | 13.0% | **-** | 3.4% |
| Co-infection with PAdV | 22.0% | 10.7% | 21.7% | 11.1% | **-** |
| Co-infection with PEDV | 23.0% | 17.9% | 39.1% | 33.3% | 10.3% |
| Co-infection with RVA | 20.0% | 19.6% | 34.8% | 11.1% | 20.7% |
| Co-infection with RVC | 1.0% | 8.9% | 0.0% | 0.0% | 3.4% |
