# Supplementary material for: Detection and genetic characterization of enteric viruses in diarrhoea outbreaks from swine farms in Spain
Source: Porcine Health Manag. 2023 Jun 22;9:29. doi: 10.1186/s40813-023-00326-w (PMC10286445; doi:10.1186/s40813-023-00326-w)
Supplement: Supplementary file 3 — Additional file 3: Summary of the results of next generation sequencing (NGS) in 14 selected faecal samples. C, consensus sequence (whole genome obtained or near complete genome, >90%); P, partial sequence (less than 90% genome covered) and T, traces (less than 10% of the genome covered). N is the number of viruses detectedper sample. An asterisk indicates PEDV genomes that were already published [8]. [file 40813_2023_326_MOESM3_ESM.docx]

**Additional file 3**: Summary of the results of next generation sequencing (NGS) in 14 selected faecal samples. C, consensus sequence (whole genome obtained or near complete genome, >90%); P, partial sequence (less than 90% genome covered) and T, traces (less than 10% of the genome covered). N is the number of viruses detected per sample. An asterisk indicates PEDV genomes that were already published [8].

| ID | Age | PAstV2 | PAstV3 | PAstV4 | PAstV5 | PKoV | PToV | PEDV | RVA | RVC | | MRV | N |
| --- | --- | --- | --- | --- | --- | --- | --- | --- | --- | --- | --- | --- | --- |
| VC4* | Nursing |  |  | P |  | C |  | C |  |  | |  | 3 |
| VC9 | Nursing | C | T | C | C | T |  | T |  |  | |  | 6 |
| VC18 | Nursing |  |  |  |  | C |  | T |  |  | |  | 2 |
| VC19 | Nursing |  |  | C | T |  | P | T |  |  | |  | 4 |
| VC29 | Postweaning | C | T | C | T | T | C | T | P | T | |  | 9 |
| VC35 | Postweaning |  | T | T |  |  |  |  | T |  | | T | 4 |
| VC36 | Fattening | C | C | C | T | C |  | T |  |  | |  | 6 |
| VC41 | Nursing | C | T | C |  | T |  |  | T | P | |  | 6 |
| VC46* | Postweaning | C | P | P | P | P | P | C |  |  | |  | 7 |
| VC57* | Nursing | T | T | C |  | T | P | C |  |  | | T | 7 |
| VC64 | Postweaning | T | T | T |  | T | T | T | T | T | |  | 8 |
| VC100* | Fattening |  | T | T |  | T |  | C | T |  | | T | 4 |
| VT9 | Fattening |  | T | C | C |  |  | T |  |  | |  | 4 |
| VT41 | Postweaning |  |  | C | T |  | P | T |  |  | |  | 4 |
|  | | | | | | | | | | |  |  |  |
|  |  |  |  |  |  |  |  |  |  |  |  |  |  |

*MRV: Mammalian orthoreovirus, PAstV: Porcine astrovirus, PEDV: Porcine epidemic diarrhoea virus, PKoV: Porcine kobuvirus, PToV: Porcine torovirus, RVA: Rotavirus A and RVC: Rotavirus C.*
